# Evaluation of the Most Stressful Dental Treatment Procedures of Conservative Dentistry among Polish Dental Students

**DOI:** 10.3390/ijerph18094448

**Published:** 2021-04-22

**Authors:** Katarzyna Mocny-Pachońska, Rafał J. Doniec, Sylwia Wójcik, Szymon Sieciński, Natalia J. Piaseczna, Konrad M. Duraj, Ewaryst J. Tkacz

**Affiliations:** 1 Department of Conservative Dentistry with Endodontics, Faculty of Medical Science, Medical University of Silesia, Pl. Akademicki 17, 41-902 Bytom, Poland; 2 Department of Biosensors and Processing of Biomedical Signals, Faculty of Biomedical Engineering, Silesian University of Technology, Roosevelta 40, 41-800 Zabrze, Poland; rafal.doniec@polsl.pl (R.J.D.); szymon.siecinski@polsl.pl (S.S.); natalia.piaseczna@polsl.pl (N.J.P.); konrad.duraj@polsl.pl (K.M.D.); etkacz@polsl.pl (E.J.T.); 3 Dental Surgery Department at the Chair and Clinic of Maxillofacial Surgery and Dental Surgery, School of Medicine with the Division of Dentistry in Zabrze, Medical University of Silesia in Katowice, Pl. Akademicki 17, 41-902 Bytom, Poland; sylwiawojcik6@poczta.onet.pl

**Keywords:** caries, dental education, endodontic treatment, stress

## Abstract

Background: Dental schools are considered to be a very stressful environment; the stress levels of dental students are higher than those of the general population. The aim of this study was to assess the level of stress among dental students while performing specific dental procedures. Methods: A survey was conducted among 257 participants. We used an original questionnaire, which consisted of 14 questions assigned to three categories: I—Diagnosis, II—Caries Treatment, and III—Endodontic Treatment. Each participant marked their perceived level of stress during the performed dental treatment procedures. The scale included values of 0–6, where 0 indicates no stress, while 6 indicates high stress. Results: Third- (p=0.006) and fourth-year (p=0.009) women were characterized by a higher level of perceived stress during dental procedures related to caries treatment. Caries treatment procedures were the most stressful for 18.3% of third-year students, 4.3% of fourth-year students, and 3.2% of fifth-year students. Furthermore, 63.4% of third-year students, 47.3% of fourth-year students, and 17.2% of fifth-year students indicated that they felt a high level of stress when performing endodontic procedures. Conclusion: Third- and fourth-year female students are characterized by a higher level of stress during caries and endodontic treatment procedures. The most stressful treatments for participants were endodontic treatment procedures.

## 1. Introduction

Stress is defined as the body’s reaction to a change that causes a physical, mental, or emotional response and can be positive and stimulating and motivate individuals to act. In some situations, however, stress may negatively affect and limit the activity of individuals [1]. Individual perceptions of stress vary widely and can be influenced by beliefs, attitudes, and occupation, among other factors. Health sciences curricula have been related to a high level of stress in students. Dental schools are considered to be a very stressful environment, and the stress levels of dental students are higher than those of the general population [2,3,4,5].

Dental studies are characterized by an extensive program that requires significant effort and a predisposition to clinical work with patients. Many factors play a role in the mental development of a dental student, one of the most important being the ability to deal with stress. Stress factors include competition, comparisons between students, teacher/student relationships, patient/student relationships, clinical application of the theory of knowledge, and individual skills. These factors can significantly influence a student’s confidence and influence the way that they perceive and experience their education [1,6].

In pre-clinical or clinical dental education, stress is ubiquitous. To reduce these feelings, especially during the transition period between pre-clinical and clinical activities, teaching assistants and lecturers need to know how to support students. Clinical training can particularly affect the performance of students due to their exposure to a variety of patient-related stressors that are similar to those experienced by dentists [7,8].

This stress also depends on the type of procedures that students perform during their training. The number of procedures performed by students during the entire three-year clinical course necessary to pass the final exam for the Conservative Dentistry with Endodontics program at the Medical University of Silesia consists of 35 restorations, 6 root canal treatments ending with obturation, and 10 examinations (including diagnosis and treatment plan). Restorations include the treatment of dental cavities, described in Black Caries Classification (i.e., classes I, II, III, IV, and V) [9,10], using composite materials.

Endodontic treatment involves the preparation of root canals using the step-back method and filling them with the lateral condensation technique [11]. The number of required root canals to be filled does not meet the requirements of the European Society of Endodontology of 2001, which recommends that the student should feel confident in their subsequent work after filling in 20 root canals. The guidelines published in 2013 were changed to emphasize the quality of endodontic treatment [12]. Nevertheless, Honey et al. showed that 81% of students from 48 dental schools in the European Union had completed 8–30 root canals (17 on average) [13].

The anatomical variety of root canals, the need to provide adequate patient care, and the lack of self-confidence among students have prompted many of them to consider endodontics to be an extremely difficult and stressful discipline. In this area, it is extremely difficult for students to translate the outcome of pre-clinical training into successful clinical treatment in a real patient. The difficulties in the initial stage of endodontic treatment often lead to frustration and dissatisfaction with pre-clinical training [8,14,15].

The aim of this study was to assess the level of stress while performing specific dental procedures among dental students in three different years of study. The second aim of the study was to confirm the hypothesis that the level of perceived stress decreases with experience, while the third aim of the study was to identify the most stressful dental procedures for students, depending on their year of study and gender.

## 2. Material and Methods

### 2.1. Experiment Setup

A total of 360 dental students of the Faculty of Medical Sciences of the Medical University of Silesia (Zabrze, Poland) were invited to take part in the study. The choice of students, which represented the third, fourth, and fifth years, was based on the program curriculum. The study was conducted between 7 and 31 January 2020. In total, 257 students completed the study.

Table 1 presents the demographic description of the study group. The $\chi^{2}$ non-parametric test of independence showed no statistically significant differences between the age groups, regardless of the respondents’ gender ($\chi^{2}=3.703$, p=0.157).

Each participant filled out a questionnaire, which consisted of 14 questions. The questions were assigned to three categories: I—Diagnosis, II—Caries Treatment, and III—Endodontic Treatment.

Category I (Diagnosis) included three questions on making a diagnosis, performing a dental examination, and taking a complete medical history. Category II (Caries Treatment) included five questions on infiltration and conductive anesthesia, placement of a matrix, and the preparation and adjustment of the filling to the occlusion. Category III (Endodontic Treatment) included six questions about cleaning of a deep cavity, endodontic access (trepanation, amputation), finding the canal orifices, measuring the working length, filling the canal, and radiological assessment of the filled canal. Each of the participants in the study marked the level of stress they felt during the performed dental treatment procedures. The blank questionnaire is provided in the Supplementary Materials.

The scale of levels of stress was based on a 7-point Likert scale (0, no stress; 6, high stress) [16]. The scale included values from 0 to 6, where 0 indicated stress free, 1 and 2—low stress, 3 and 4—medium stress, and 5 and 6—high stress. The questionnaire was filled in during seminar classes in accordance with the schedule of the given student group. All participants were provided with the same conditions: no more than 10 people in the room, a room insulated from noise, adequately lighting and ventilation, sufficient space to complete the survey, and comfortable sitting.

The study was approved by the Ethical Commission of Medical University of Silesia, under the resolution number KNW/0022/KB1/79/18 taken on 16 October 2018. All participants gave informed consent before inclusion in the study group.

### 2.2. Statistical Analysis

Statistical analysis was performed using the Statistica Version 9.0 (StatSoft, Inc., Tulsa, OK, USA) and Microsoft Excel (Microsoft Corporation, Redmond, WA, USA) software. Due to the significant differences between the students representing different consecutive years of study and gender, we used the non-parametric $\chi^{2}$ test of independence. The Mann–Whitney test was used to assess the significance of differences in the scale values between genders for the subsequent years of study. The significance of gender differences in the subsequent years of study was assessed by the Kruskal–Wallis test. The statistical analysis was supported by post hoc tests in two variants: Dunn’s test (Dunn–Bonferroni test) and Conover test. The level of statistical significance (*p*) was set to 0.05.

## 3. Results

### 3.1. Reliability of the Scale

To examine the reliability of proposed stress scales, we calculated the Cronbach’s alpha coefficients; for which an a value over 0.7 suggests high reliability [17]. Table 2 presents the Cronbach’s alpha coefficients.

The values presented in Table 2 prove the high reliability of the proposed stress scale. The elimination of Question 3 (on making a diagnosis) slightly improved the reliability of the stress scale, similarly to the removal of Question 6. The question on the treatment of deep cavity group slightly improved the Cronbach’s alpha coefficient.

### 3.2. Category I (Diagnosis)

**Figure 1:**
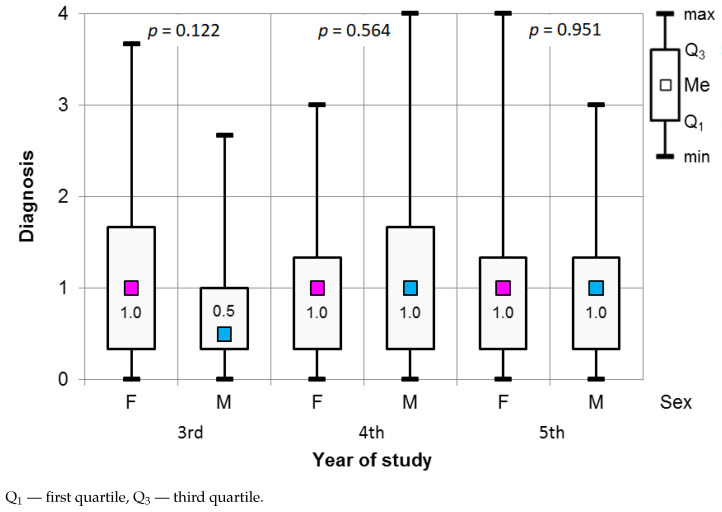
Results of the Mann–Whitney test used to assess the significance of differences in the Diagnosis procedures in relation to gender and year of study.

**Figure 2:**
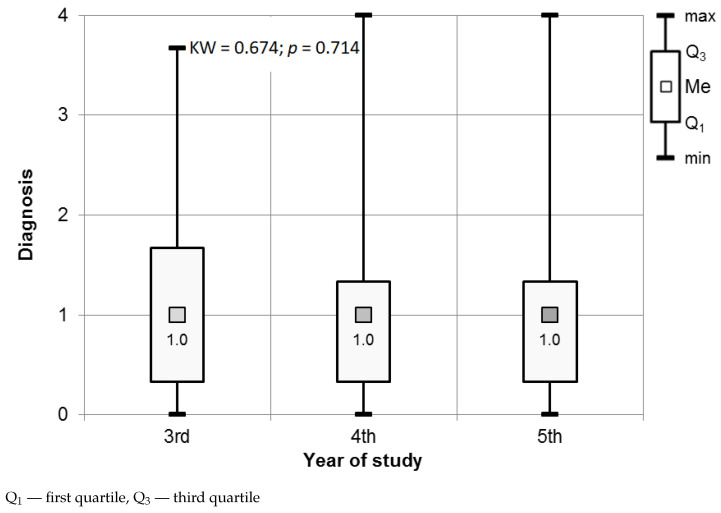
Results of the Kruskal–Wallis test used to assess the significance of differences in the Diagnosis procedures in relation to gender and year of study.

The results of the Mann–Whitney test showed insignificant differences between genders (see Figure 1) for Diagnosis procedures. We did not observe any significant differences in the stress level related to Diagnosis procedures between the participants as related to the year of study (see Figure 2).

**Figure 3:**
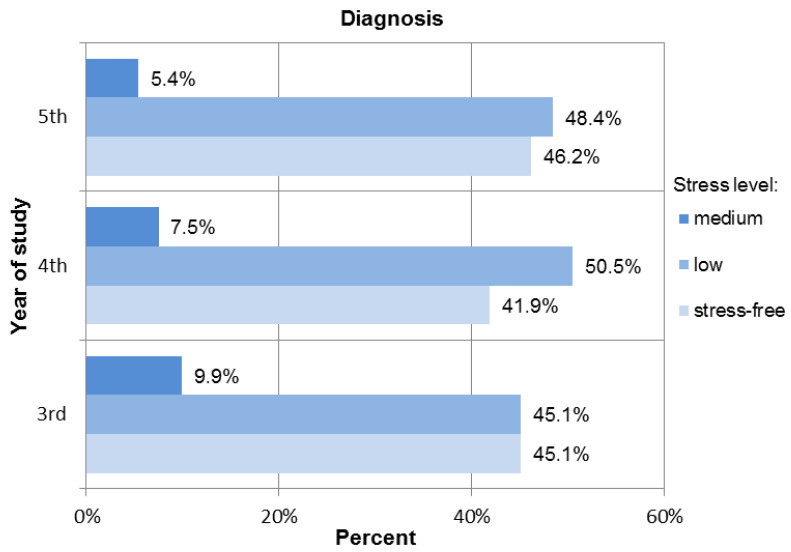
Distribution of the stress levels among students for Diagnosis procedures.

No participant reported a high level of perceived stress when answering the questions relating to diagnosis. The responses indicated that 45.1% of the third-year students, 41.9% of the fourth-year students, and 46.2% of the fifth-year students felt no stress during the dental examination, interview, and diagnosis procedures (see Figure 3).

### 3.3. Category II (Caries Treatment)

**Figure 4:**
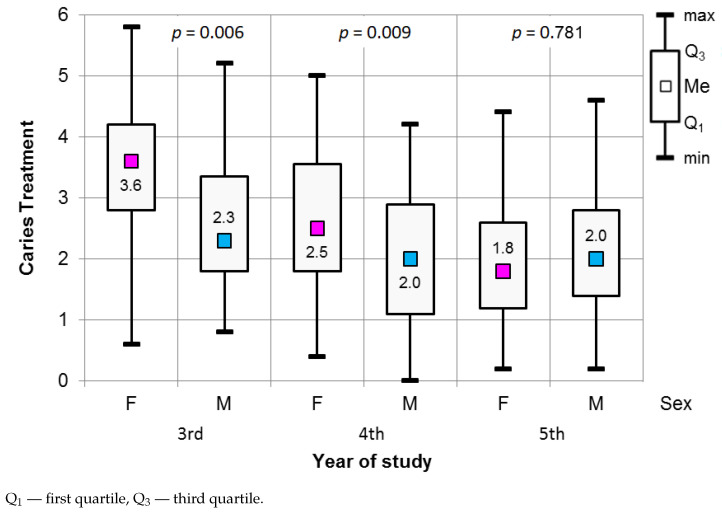
Results of the Mann–Whitney test used to assess the significance of differences in the Caries treatment procedures in relation to gender and year of study.

The results of the Mann–Whitney test used to assess the significance of the differences in the values for the stress scale in Caries Treatment are presented in Figure 4. The differences between men and women in their third (p=0.006) and fourth years of study were statistically significant (p=0.009).

In both years of study, women were characterized by a higher level of perceived stress during dental procedures related to caries treatment. The median for women in their third year was 3.60, while that for men was 2.30. In the case of fourth-year students, these values were 2.50 for women and 2.0 for men (see Figure 4).

**Figure 5:**
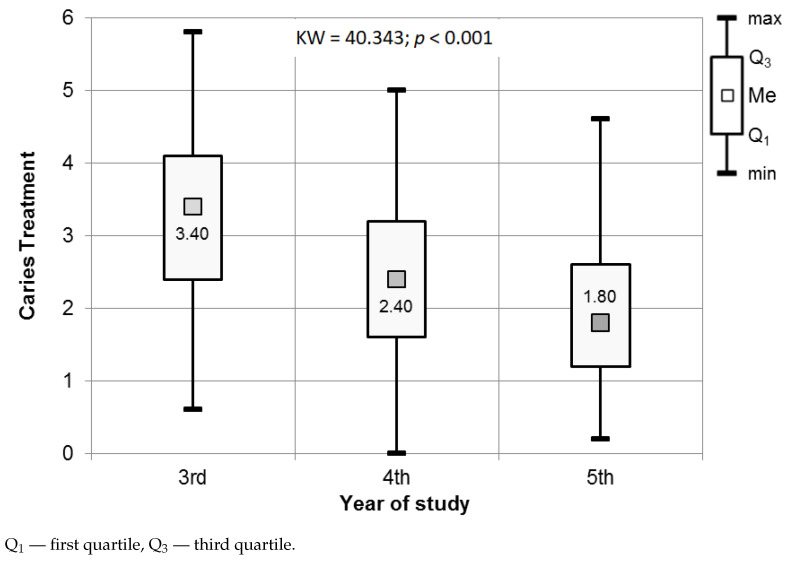
Results of the Kruskal–Wallis test used to assess the significance of differences in the Caries treatment procedures in relation to the year of study.

The most prominent differences in perceived stress were observed for the questions covering the caries treatment, which were proved by the Kruskal–Wallis test (p<0.001) performed on participants representing each analyzed year of study. Complementary post hoc tests confirmed the significant differences in the level of perceived stress between the participants of each analyzed year (Dunn’s test, p<0.001), third and fifth year (Dunn’s test, p<0.001), and fourth and fifth year (Dunn’s test, p=0.03). In all cases, lower-year students were characterized by a higher level of perceived stress (see Figure 5, Table 3).

**Figure 6:**
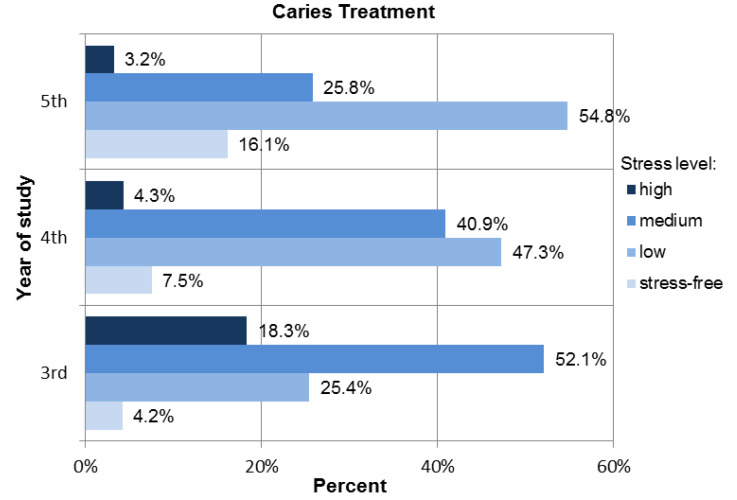
Distribution of stress levels among students for Caries treatment procedures.

A total of 18.3% of third-year students, 4.3% of fourth-year students, and 3.2% of fifth-year students experienced high levels of stress related to caries treatment procedures; 54.8% of students in their fifth year experienced low levels of stress. In the case of third-year students, the dominant level of perceived stress was moderate (52.1%). Fourth-year participants experienced low stress in 47.3% of cases (see Figure 6).

### 3.4. Category III (Endodontic Treatment)

**Figure 7:**
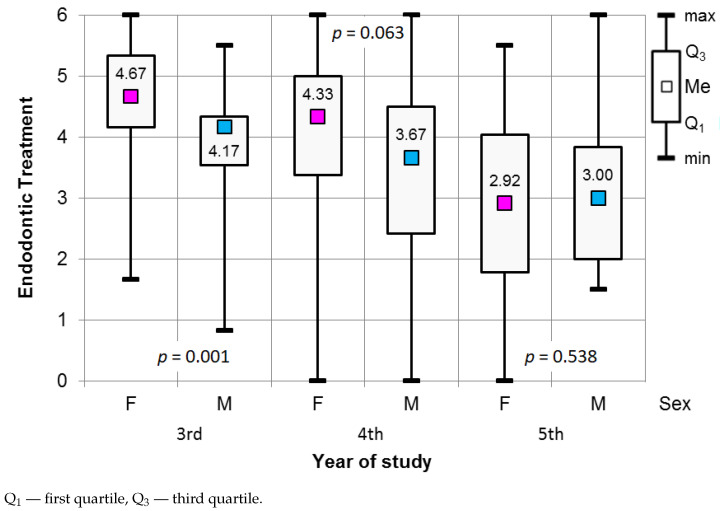
Results of the Mann–Whitney test used to assess the significance of differences in the Endodontic treatment procedures in relation to gender and year of study.

Endodontic treatment procedures were the most stressful for third-year participants. Women in their third year of study perceived a higher level of stress (median of 4.6) than men in the same year of study (median of 4.17), with a significance of p=0.001. The results of the Mann–Whitney test, used to assess the significance of the differences in the values for the stress scale in Endodontic treatment, are presented in Figure 7. The difference in perceived stress level between men (median of 3.67) and women (median of 4.33) representing the fourth year of study was statistically significant (p=0.063), as shown in Figure 7.

**Figure 8:**
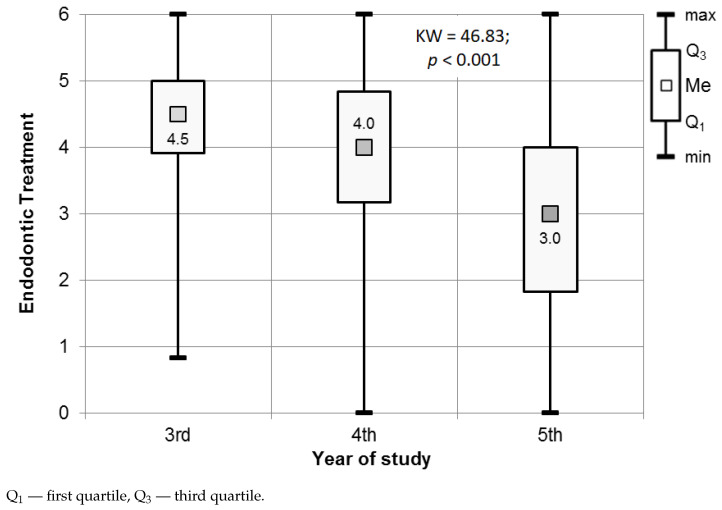
Results of the Kruskal–Wallis test used to assess the significance of differences in the Endodontic treatment procedures in relation to the year of study.

The results of the Kruskal–Wallis test (*p* < 0.001), supplemented with post hoc tests, demonstrated significant differences in the perceived level of stress during endodontic procedures between third- and fourth-year students (Dunn’s test, p=0.04), third- and fifth-year students (Dunn’s test, p<0.001), and between fourth- and fifth-year students (Dunn’s test, p<0.001). The fifth-year students (median of 3) were stressed the least when performing endodontic procedures (see Figure 8, Table 4).

**Figure 9:**
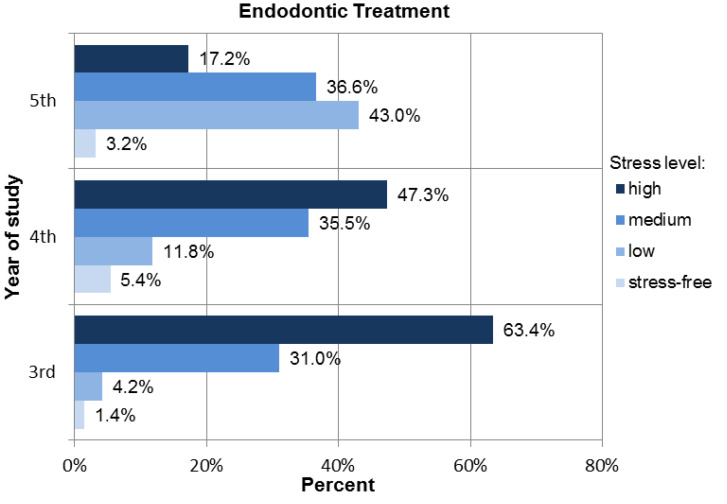
Distribution of the stress levels among students for Endodontic treatment procedures.

Endodontic treatment procedures turned out to be the most stressful treatment procedures for students: 63.4% of third-year students, 47.3% of fourth-year students, and 17.2% of fifth-year students indicated that they felt a high level of stress when performing endodontic procedures. Moreover, moderate stress was experienced by 31% of third-year students, 35.5% of fourth-year students, and 36.6% of fifth-year students (see Figure 9).

## 4. Discussion

Dental studies programs require students to master theoretical knowledge and to attend practical training sessions [18]. For many of them, the significant amount of theoretical knowledge and practical skills to master increases the level of perceived stress, especially for those with a lower level of manual skills. Many procedures performed during dental treatment are very demanding, such as endodontic treatment.

Tanalp et al., when assessing a group of 48 fifth-year students, discovered that 11.9% found endodontic procedures to be very difficult. The students felt the least confident in inserting a rubber dam, in whitening endodontically treated teeth, in the treatment of resorption, and with endo-perio cases, as well as in endodontic apexification and retreatment processes [12]. In our findings, 17.2% of the 93 fifth-year students who completed the questionnaire experienced high levels of stress related to endodontic treatment.

Meanwhile, 36.6% of the fifth-year students experienced an average level of stress. The questions did not mention complicated procedures, such as endodontic retreatment or resorption treatment—these procedures are not performed by students, according to the program curriculum at the Medical University of Silesia. The questions were related to the basic methods of treatment, including endodontic access, finding the root canal orifices, measuring the working length and filling the canal, and radiological evaluation after the end of treatment. Despite this fact, 53.8% of fifth-year students experienced moderate to high levels of stress. In the case of fourth-year students, 47.3% of them experienced a high level of stress during these procedures, while 63.4% of third-year students experienced high stress levels.

Tavares et al. observed that over 60% of 102 students who took pre-clinical or clinical classes in endodontics did not report any difficulties with proper endodontic access. On the other hand, approximately 44% of students in the pre-clinical endodontics phase reported difficulties with the correct performance of the crown-down technique, although the number of students reporting difficulties with this method decreased to 4% during the clinical course. A low percentage of students reported problems with determining the working length and choosing the correct instrumentation. Most problems were experienced by the stutters when the appropriate medication was prescribed during multi-session root canal treatment. Most students, however, confirmed that they were able to overcome the aforementioned difficulties, thanks to further education and experience [19].

According to the study of Alrahabi, the differences in the level of self-confidence between students in their fourth and fifth years of study were statistically significant, in favor of fifth-year students, with regard to the following procedures: the determination of the working length, the interpretation of radiographic imagery, the assessment of canal fillings, and setting follow-up visits [20]. In our study, the differences in the level of perceived stress related to the performance of endodontic procedures between students in their fourth and fifth year of study were statistically significant, where fifth-year students reported less stress. Female fourth-year students perceived a higher level of stress than their male counterparts.

Difficulties in performing endodontic procedures have also been noted in [7,20,21,22,23]. Another important aspect considered in the study was the transition of the theoretical knowledge and practical skills from the pre-clinical phase to the clinical environment, which requires contact with real patients. This was also confirmed in our study, where the highest level of stress was observed in third-year students in comparison with fourth- and fifth-year students [3].

Among the results, it can also be noted that female third-year students felt higher levels of stress than their male counterparts. This is probably due to the fact that the third year is when students start working in a clinical setting. In their study, Frese et al. showed that the level of perceived stress is clearly higher among students starting clinical classes, especially in the first weeks of these classes. According to Frese et al. [8], the most stressful procedures are endodontic treatment followed by restorative and periodontal treatment. Reported stress has also been associated with a negative impact on psychosocial interactions.

The main purpose of the course is to provide the relevant knowledge and skills to work efficiently as a dentist. Hattar et al. showed that fifth-year students felt most confident while performing direct restorations and simple endodontic treatment. However, they experienced difficulties while performing complex endodontic treatment and indirect restorations [24].

In our study, Polish fifth-year students perceived significantly lower levels of stress when performing procedures related to diagnosis, caries treatment, and endodontic treatment than third- and fourth-year students. In the case of caries treatment, over 50% of them experienced a low level of stress, while 16.1% did not find the procedures stressful. Similar results on caries treatment have been obtained by Rajan et al., where high confidence was experienced by undergraduate students for caries management and preventive care, while the lowest confidence was reported for the management of oral medicine, pathologies, and dental emergencies [25].

The levels of perceived stress and confidence among fourth- and fifth-year students during the preparation of cavities from deep caries and possible pulp exposure depend on the performed procedure, academic education, clinical training, the equipment and organization of the clinical room, vital pulp material, and the patient. In order to reduce stress, students have indicated the need to implement modern teaching methods (e.g., the use of 3D printers) and to develop the same algorithms of conduct among teachers [14].

## 5. Limitation of the Study

This study raises very important issues related to the dental education and the transfer of skills attained at university to the professional career. Nevertheless, it does come with some limitations. The study was conducted among students of only one university (Medical University of Silesia). Such a comparison between students of several Polish universities could give a broader perspective. Including more students in the study would certainly also strengthen the statistical analyses. These factors could affect any changes to the curriculum more significantly.

## 6. Conclusions

Women in their third year of study were characterized by a higher level of perceived stress compared to men in the same year of study. Third-year participants experienced the greatest stress in the case of caries treatment and endodntic treatment procedures. The obtained results confirmed the hypothesis that the level of perceived stress decreases with the experience acquired. We confirmed that endodontic treatment procedures are the most stressful procedures for students, regardless of their year of study. Emphasis on increased education in this direction should be included in the curriculum.

## 7. Practical Application

The information about the most stressful procedures for students should lead to underlining the importance of preparing the students for performing them in clinical practice. Our study showed that the the transfer of skills from the pre-clinical to the clinical phase is an important part of the dental studies curriculum. The highest level of stress was experienced by the third-year students, who had just begun classes in a clinical setting. Using medical simulation techniques could improve the outcomes of the pre-clinical phase by preparing the students to perform conservative dentistry procedures on patients in clinical conditions.

## Figures and Tables

**Table 1:** Demographic data of the participants (dental students at the Medical University of Silesia).

Year of Study	Number of Students	Median Age [Years]
Third	71	23
	(53 women, 18 men)	
Fourth	93	24
	(58 women, 35 men)	
Fifth	93	24
	(68 women, 25 men)	

**Table 2:** Analysis of scale reliability.

Survey					Item Elimination	
Group		α	$\alpha_{\mathrm{st}}$	$r_{a}v$	Item	α
I (Diagnosis)	Total	0.75	0.80	0.56	3	0.76
	Female	0.73	0.77	0.53	3	0.74
	Male	0.80	0.84	0.64	3	0.81
II (Caries Treatment)	Total	0.82	0.81	0.47	-	-
	Female	0.81	0.80	0.45	-	-
	Male	0.82	0.82	0.45	-	-
III (Endodontic Treatment)	Total	0.89	0.89	0.59	6	0.91
	Female	0.89	0.90	0.60	6	0.91
	All	0.88	0.89	0.57	6	0.90

*α*—Cronbach’s alpha, *α_st_*—standardized alpha, *r_av_*—average correlation coefficient.

**Table 3:** Results of post hoc tests—Caries treatment procedures.

	Dunn–Bonferroni			
Conover	Year of study	Third	Fourth	Fifth
	Third		<0.001	<0.001
	Fourth	0.05		0.03
	Fifth	0.02	NS	

NS: Not significant.

**Table 4:** Results of post hoc tests—Endodontic treatment procedures.

	Dunn-Bonferroni			
Conover	Year of study	Third	Fourth	Fifth
	Third		0.04	<0.001
	Fourth	NS		<0.001
	Fifth	0.02	0.04	

NS: Not significant.

## Data Availability

The data presented in this study are available on a reasonable request from the corresponding author.

## Supplementary Materials

The following are available online at https://www.mdpi.com/article/10.3390/ijerph18094448/s1, Questionnaire S1: What level of stress do you feel when performing the following procedures.
