# Supplementary material for: Evaluation of the Most Stressful Dental Treatment Procedures of Conservative Dentistry among Polish Dental Students
Source: Int J Environ Res Public Health. 2021 Apr 22;18(9):4448. doi: 10.3390/ijerph18094448 (PMC8122741; doi:10.3390/ijerph18094448)
Supplement: Supplementary file 1 [file ijerph-18-04448-s001.zip › ijerph-1160783-supplementary.pdf]

**What level of stress do you feel when performing the following procedures**		
Year of study:	Age:	Sex: Female / Male
**Scale: 0 1 2 3 4 5 6**<br>0 – no stress; 1, 2 – low stress; 3, 4 – medium stress; 5, 6 – high stress.		

1. Determination of working lenght (III,12)	0 1 2 3 4 5 6
2. Treatment of a deep caries (II,6)	0 1 2 3 4 5 6
3. Making a diagnosis (I, 3)	0 1 2 3 4 5 6
4. Finding the canals‘ orifiices (III, 11)	0 1 2 3 4 5 6
5. Adjustment of the filling to the occlusion (II, 8)	0 1 2 3 4 5 6
6. Radiological evaluation of the filled canal (III, 14)	0 1 2 3 4 5 6
7. Infiltration anesthesia (II, 4)	0 1 2 3 4 5 6
8. Pulpotomy (III,10)	0 1 2 3 4 5 6
9. Placement of a matrix (II, 7)	0 1 2 3 4 5 6
10. Dental examination (I, 2)	0 1 2 3 4 5 6
11. Prepating endodontic access (III, 9)	0 1 2 3 4 5 6
12. Conductive anesthesia(II, 5)	0 1 2 3 4 5 6
13. Taking patient’s anamnesis (I, 1)	0 1 2 3 4 5 6
14. Oburation of a root canal (III, 13)	0 1 2 3 4 5 6

I-Diagnosis

II Caries Treatment

III Endodotic Treatment
